# Intervention in prediction measure: a new approach to assessing variable importance for random forests

**DOI:** 10.1186/s12859-017-1650-8

**Published:** 2017-05-02

**Authors:** Irene Epifanio

**Affiliations:** Departament de Matemàtiques and Institut de Matemàtiques i Aplicacions de Castelló, Universitat Jaume I, Campus del Riu Sec, Castelló, 12071 Spain

**Keywords:** Random forest, Variable importance measure, Multivariate response, Feature selection, Conditional inference trees

## Abstract

**Background:**

Random forests are a popular method in many fields since they can be successfully applied to complex data, with a small sample size, complex interactions and correlations, mixed type predictors, etc. Furthermore, they provide variable importance measures that aid qualitative interpretation and also the selection of relevant predictors. However, most of these measures rely on the choice of a performance measure. But measures of prediction performance are not unique or there is not even a clear definition, as in the case of multivariate response random forests.

**Methods:**

A new alternative importance measure, called Intervention in Prediction Measure, is investigated. It depends on the structure of the trees, without depending on performance measures. It is compared with other well-known variable importance measures in different contexts, such as a classification problem with variables of different types, another classification problem with correlated predictor variables, and problems with multivariate responses and predictors of different types.

**Results:**

Several simulation studies are carried out, showing the new measure to be very competitive. In addition, it is applied in two well-known bioinformatics applications previously used in other papers. Improvements in performance are also provided for these applications by the use of this new measure.

**Conclusions:**

This new measure is expressed as a percentage, which makes it attractive in terms of interpretability. It can be used with new observations. It can be defined globally, for each class (in a classification problem) and case-wise. It can easily be computed for any kind of response, including multivariate responses. Furthermore, it can be used with any algorithm employed to grow each individual tree. It can be used in place of (or in addition to) other variable importance measures.

**Electronic supplementary material:**

The online version of this article (doi:10.1186/s12859-017-1650-8) contains supplementary material, which is available to authorized users.

## Background

High-dimensional problems, those that involve so-called *p*>*n* data [1], are of great importance in many areas of computational biology. Predicting problems in which the number of features or variables *p* is much larger than the number of samples or observations *n* is a statistical challenge. In addition, many bioinformatics data sets contain highly correlated variables with complex interactions, and they may also contain variables that are irrelevant to the prediction. Furthermore, data sets may contain data of a mixed type, i.e. categorical (with a different number of categories) and numerical, not only as predictors but also as outputs or responses.

Decision trees are a nonparametric and highly nonlinear method that can be used successfully with that kind of challenging data. Furthermore, they are robust to outliers in the input space, invariant to monotone transformations of numerical predictors, and can also handle missing values. Thanks to these properties, decision trees have become a very popular tool in bioinformatics and data mining problems in general.

However, the predictive power of decision trees is their Achilles heel. A bagging strategy can be considered to improve their individual performance. A random forest (RF) is an ensemble of a large collection of trees [2]. There are several types of RFs according to the type of response. If the response is categorical, we refer to RF classification. If the response is continuous, we refer to RF regression. If the responses are right censored survival data, we refer to Random Survival Forests. Multivariate RFs refer to RFs with multiple responses [3]. RFs with only one response are applied to many different problems in bioinformatics [4, 5]. However, the number of studies with multivariate RFs is much smaller [6].

Besides good performance, another advantage of RFs is that they require little tuning. Another property of RFs that makes them attractive is that they return variable importance measures (VIMs). These VIMs can be used to rank variables and identify those which most influence prediction. This favors interpretability. Predictors are not usually equally relevant. In fact, often only a few of them have a substantial influence on the response, i.e. the rest of them are irrelevant and could have been excluded from the analysis. It is often useful to learn the contribution or importance of explanatory variables in the response [1].

The most widely used VIMs for RFs, such as the Gini VIM (GVIM), permutation VIM (PVIM) and conditional permutation VIM (CPVIM) [7], rely on the choice of a performance measure. However, measures of prediction performance are not unique [8]. Some examples for classification are misclassification cost, Brier score, sensitivity and specificity measures (binary problems), etc., while some examples for regression problems are mean squared error, mean absolute error, etc. In the case of unbalanced data, i.e. data where response class sizes differ considerably, the area under the curve (AUC) is suggested by [9] instead of the common error rate. There is no clear appropriate performance measure for survival data [10], less so in the case of multivariate response and even less so if the responses are of different types.

To solve this issue, an approach for selecting variables that depends on the structure of the trees, without depending on performance measures, was proposed by [10]. They proposed an algorithm based on the minimal depth (MD) statistic, i.e. based on the idea that variables that tend to split close to the root node should have more importance in prediction. By removing the dependence on performance measures, the arrangement of the trees gains strength, as in the case of splitting rules.

Recently, the author has proposed a new alternative importance measure in RFs called Intervention in Prediction Measure (IPM) [11] in an industrial application. IPM is also based on the structure of the trees, like MD. Therefore, it is independent of any prediction performance measure. IPM only depends on the forest and tree parameter settings. However, unlike MD, IPM is a case-based measure. Note also that IPM is expressed as a percentage, which makes it attractive in terms of interpretability. IPM can be used with new observations that were not used in the RF construction, without needing to know the response, unlike other VIMs. IPM can be defined globally, for each class (in a classification problem) and locally. In addition, IPM can easily be computed for any kind of response, including multivariate responses. IPM can be used with any algorithm employed to grow each individual tree, from the Classification And Regression Trees (CART) algorithm developed by [12] to Conditional Inference Trees (CIT) [13].

The objective of this work is to compare the new IPM with other well-known VIMs in different contexts, such as a classification problem with variables of different types, another classification problem with correlated predictor variables, and problems with multivariate responses and predictors of different types. Several simulation studies were carried out to show the competitiveness of IPM. Furthermore, the objective is also to stress the advantages of using IPM in bioinformatics. Consequently, the use of IPM is also illustrated in two well-known bioinformatics applications previously employed in other papers [14–16]. Although the majority of the data used here are not *p*>*n*, they could be relevant to this kind of scenarios, but this is something to be explored in the future.

## Methods

### Random forest

As mentioned previously, trees are a nonlinear regression procedure. Trees are grown by binary recursive partitioning. The broad idea behind binary recursive partitioning is to iteratively choose one of the predictors and the binary split in this variable in order to ultimately fit a constant model in each cell of the resulting partition, which constitutes the prediction. Two known problems with such models are overfitting and a selection bias towards predictors with many possible splits or missing values [15]. To solve these problems, [13] proposed a conditional inference framework (CIT) for recursive partitioning, which is also applicable to multivariate response variables, and it will be used with IPM.

As outlined above, trees are a low-bias but high-variance technique, which makes them especially suited for bagging [1]. Growing an ensemble of trees significantly increases the accuracy. The term random forest was coined by [2] for techniques where random vectors that control the growth of each tree in the ensemble are generated. This randomness comes from randomly choosing a group of *mtry* (*mtry* <<*p*) predictors to split on at each node and bootstrapping a sample from the training set. The non-selected cases are called out-of-bag (OOB).

Here, RFs based on CART (CART-RF) and CIT (CIT-RF) are considered since the VIMs reviewed later are based on these. Both are implemented in R [17]. Breiman’s RF algorithm [2] is implemented in the R package randomForest [18, 19] and also in the R package randomForestSRC [20–22], while CIT-RF can be found in the R package party [23–25]. Multivariate RFs can be computed by the R package randomForestSRC and the R package party, but not by the R package randomForest. In the R package randomForestSRC, for multivariate regression responses, a composite normalized mean-squared error splitting rule is used; for multivariate classification responses, a composite normalized Gini index splitting rule is used; and when both regression and classification responses are detected, a multivariate normalized composite split rule of mean-squared error and Gini index splitting is invoked.

Regardless of the specific RF implementation, VIMs can be computed, which are a helpful tool for data interpretation and feature selection. VIMs can be used to obtain a ranking of the predictors according to their association with the response. In the following section, the most used VIMs are briefly reviewed and our IPM proposal is introduced.

### Random forest variable importance measures and variable selection procedures

The most popular VIMs based on RF include GVIM, PVIM and CPVIM. GVIM and PVIM are derived from CART-RF and can be computed with the R package randomForest [18, 19]. PVIM can also be derived from CIT-RF and obtained with the R package party [23–25]. CPVIM is based on CIT-RF, and can be calculated using the R package party. A very popular stepwise procedure for variable selection using PVIM is the one proposed by [26] (varSelRF), which is available from the R package varSelRF [27, 28]. The procedure for variable selection based on the tree-based concept termed MD proposed by [10] (varSelMD) is available from the R package randomForestSRC [20–22]. The results of the chosen variables for variable selection methods can be interesting, although the objective of variable selection methods is not really returning the importance of variables, but returning a set of variables that are subject to a certain objective, such as preserving accuracy in [26]. The underlying rationale is that the accuracy of prediction will not change if irrelevant predictors are removed, while it drops if relevant ones are removed. Table 1 gives an overview of the methods.

**Table 1:** Summary of some characteristics of VIMs

Methods	GVIM	PVIM (CART-RF)	PVIM (CIT-RF)	CPVIM	varSelRF	varSelMD	IPM
Main references	[43]	[2]	[13, 15]	[25]	[26]	[10, 33]	[11] and this manuscript
Key characteristic	Node impurity	Accuracy after variable permutation	Accuracy after variable permutation	Alternative of PVIM; Conditional permutation	Backward elimination	Variable selection based on MD	Variables intervening in prediction
RF-based	CART-RF	CART-RF	CIT-RF	CIT-RF	CART-RF	CART-RF	CART-RF or CIT-RF
Handling of response	Univariate	Univariate	Univariate	Univariate	Categorical	All (multivariate included)	All (multivariate included)
Main R implementation	randomForest [18, 19]	randomForest [18, 19] randomForestSRC [20–22]	party [23–25]	party [23–25]	varSelRF [27, 28]	randomForestSRC [20–22]	Additional file 2
Casewise importance	No	Yes	Not defined	Not defined	No	No	Yes

#### GVIM

GVIM is based on the node impurity measure for node splitting. The node impurity is measured by the Gini index for classification and by the residual sum of squares for regression. The importance of a variable is defined as the total decrease in node impurities from splitting on the variable, averaged over all trees. It is a global measure for each variable; it is not defined locally or by class (for classification problems). When there are different types of variables, GVIM is strongly biased in favor of continuous variables and variables with many categories (the statistical reasons for this are well explained by [15]).

#### PVIM

The importance of variable *k* is measured by averaging over all trees the decrease in accuracy between the prediction error for the OOB data of each tree and the same after permuting that predictor variable.

The PVIM derived from CIT-RF is referred to as PVIM-CIT-RF. Although CIT-RF can fit multivariate responses, PVIM cannot be computed (as previously discussed, there is no clear appropriate performance measure for multivariate responses).

The PVIM derived from CART-RF is referred to as PVIM-CART-RF, which is scaled (normalized by the standard deviation of the difference) by default in the randomForest function from the R package randomForest [18, 19]. The problems of this scaled measure are explored by [29]. According to [30], PVIM is often very consistent with GVIM. PVIM can be computed for each class, and it can also be computed casewise. The local or casewise variable importance is the increase in percent of times a case *i* is OOB and misclassified when the variable *k* is permuted. This option is available from the R package randomForest [18, 19], but not from the R package party [23–25].

#### CPVIM

An alternative version of PVIM to correct bias for correlated variables is CPVIM, which uses a conditional permutation scheme [25]. According to [31], CPVIM would be more appropriate if the objective is to identify a set of truly influential predictors without considering the correlated effects. Otherwise, PVIM would be preferable, as correlations are an inherent mutual property of predictors. In CPVIM, the variable importance of a predictor is computed conditionally on the values of other associated/correlated predictor variables, i.e. possible confounders are taken into account, unlike PVIM. The concept of confounding is well illustrated with a simple example considered in [32] (see [32] for a more extensive explanation): a classification problem for assessing fetal health during pregnancy. Let *Y* be the response with two possible values (*Y*=0 if the diagnosis is incorrect and *Y*=1 otherwise). Let us consider the following predictors: *X*_1_, which assesses the quality of ultrasound devices in the hospital, *X*_2_, which assesses whether the hospital staff are trained to use them and interpret the images and *X*_3_, which assesses the cleanliness of hospital floors. Note that *X*_2_ is related to *Y* and *X*_3_, which are linked to the hospital’s quality standards. If *X*_2_ was not taken into account in the analysis, a strong association between *Y* and *X*_3_ would probably be found, i.e. *X*_2_ would act as a confounder. In the multiple regression model, if *X*_2_ was included as predictor in the model, the questionable influence of *X*_3_ would disappear. This is the underlying rationale for CPVIM: conditionally on *X*_2_, *X*_3_ does not have any effect on *Y*.

#### varSelRF

Díaz-Uriarte and Alvarez de Andrés [26] presented a backward elimination procedure using RF for selecting genes from microarray data. This procedure only applies to RF classification. They examine all forests that result from iteratively eliminating a fraction (0.2 by default) of the least important predictors used in the previous iteration. They use the unscaled version of PVIM-CART-RF. After fitting all forests, they examine the OOB error rates from all the fitted random forests. The OOB error rate is an unbiased estimate of the test set error [30]. They select the solution with the smallest number of genes whose error rate is within 1 (by default) standard error of the minimum error rate of all forests.

#### varSelMD

MD assesses the predictiveness of a variable by its depth relative to the root node of a tree. A smaller value corresponds to a more predictive variable [33]. Specifically, the MD of a predictor variable *v* is the shortest distance from the root of the tree to the root of the closest maximal subtree of *v*; and a maximal subtree for *v* is the largest subtree whose root node is split using *v*, i.e. no other parent node of the subtree is split using *v*. MD can be computed for any kind of RF, including multivariate RF.

A high-dimensional variable selection method based on the MD concept was introduced by [10]. It uses all data and all variables simultaneously. Variables with an average MD for the forest that exceeds the mean MD threshold are classified as noisy and are removed from the final model.

### Intervention in prediction measure (IPM)

IPM was proposed in [11], where an RF with two responses (an ordered factor and a numeric variable) was used for child garment size matching.

IPM is a case-wise technique, i.e. IPM can be computed for each case, whether new or used in the training set. This is a different perspective for addressing the problem of importance variables.

The IPM of a new case, i.e. one not used to grow the forest and whose true response does not need to be known, is computed as follows. The new case is put down each of the *ntree* trees in the forest. For each tree, the case goes from the root node to a leaf through a series of nodes. The variable split in these nodes is recorded. The percentage of times a variable is selected along the case’s way from the root to the terminal node is calculated for each tree. Note that we do not count the percentage of times a split occurred on variable *k* in tree *t*, but only the variables that intervened in the prediction of the case. The IPM for this new case is obtained by averaging those percentages over the *ntree* trees. Therefore, for IPM computation it is only necessary to know the structure of the trees forming the forest; the response is not necessary.

The IPM for a case in the training set is calculated by considering and averaging over only the trees where the case belongs to the OOB set. Once the casewise IPMs are estimated, the IPM can be computed for each class (in the case of RF-classification) and globally, averaging over the cases in each class or all the cases, respectively. Since it is a case-wise technique, it is also possible to estimate the IPM for subsets of data, with no need to regrow the forest for those subsets.

An anonymous reviewer raised the question of using in-sample observations in the IPM estimation. In fact, the complete sample could be used, which would increase the sample size. This is a matter for future study. Although IPM is not based on prediction, i.e. it does not need the responses for its computation once the RF is built, the responses of in-sample observations were effectively used in the construction of the trees. So brand new and unused data (OOB observations) were preferred for IPM estimation, in order to ensure generalization. In Additional file 1, there is an example using all samples.

The new IPM and all of the code to reproduce the results are available in Additional file 2.

### Comparison studies

The performance of IPM in relation to the other well-established VIMs is compared in several scenarios, with simulated and real data. Two different kinds of responses are analyzed with both simulated and real data, specifically RF-classification and Multivariate RF are considered in order to cover the broadest possible spectrum of responses.

The importance of the variables is known a priori with simulated data, as we know the model which generated the data. In this way, we can reliably analyze the successes in the ranking and variable selection for each method, and also the stability of the results, as different data sets are generated for each model. For RF-classification, the simulation models are analogous to those considered in previous works. For Multivariate RFs, simulation models are designed starting from scratch in order to analyze their performance under different situations.

Analyses are also conducted on real data, which have previously been analyzed in the literature in order to supply additional evidence based on realistic bioinformatics data structures that usually incorporate complex interdependencies.

Once importance values are computed, predictors can be ranked in decreasing order of importance, i.e. the most important variable appears in first place. For some methods there are ties (two variables are equally important). In such cases, the average ranking is used for those variables.

All the computations are made in R [17]. The packages and parameters used are detailed for each study.

### Simulated data

#### Categorical response: Scenarios 1 and 2

Two classification problems are simulated. In both cases, a binary response *Y* has to be predicted from a set of predictors.

In the first scenario, the simulation design was similar to that used in [15], where predictors varied in their scale and number of categories. The first predictor *X*_1_ was continuous, the other predictors from *X*_2_ to *X*_5_ were categorical with a different number of categories. Only predictor *X*_2_ intervened in the generation of the response *Y*, i.e. only *X*_2_ was important, the other variables were uninformative, i.e. noise. This should be reflected in the VIM results. The simulation design of Scenario 1 appears in Table 2. The number of cases (predictors and response) generated in each data set was 120. A total of 100 data sets were generated, so the stability of the results could also be assessed.

**Table 2:** Simulation design for Scenario 1

Variables	*X*_1_	*X*_2_	*X*_3_	*X*_4_	*X*_5_	*Y*|*X*_2_=0	*Y*|*X*_2_=1
Distribution	N(0,1)	B(1,0.5)	DU(1/4)	DU(1/10)	DU(1/20)	B(1,0.5 - *rel*)	B(1,0.5 + *rel*)

The variables are sampled independently from the following distributions. N(0,1) stands for the standard normal distribution. B(1, *π*) stands for the Binomial distribution with *n* = 1, i.e the Bernoulli distribution, and probability *π*. DU(1/*n*) stands for the Discrete Uniform distribution with values 1, …, *n*. The relevance parameter *rel* indicates the degree of dependence between *Y* and *X*_2_, and is set at 0.1, which is not very high

The parameter settings for RFs were as follows. CART-RF was computed with bootstrap sampling without replacement, with *n* *t* *r* *e* *e*=50 as in [15], and two values for *mtry*: 2 (sqrt(*p*) the default value in [18]) and 5 (equal to *p*). GVIM, PVIM and IPM were computed for CART-RF. CIT-RF was computed with the settings suggested for the construction of an unbiased RF in [15], again with *ntree* =50 and *mtry* equal to 2 and 5. PVIM, CPVIM, and IPM were computed for CIT-RF. varSelRF [27] was used with the default parameters (*ntree* =5000). varSelMD [20] was used with the default parameters (*ntree* =1000 and *m* *t* *r* *y*=2) and also with *mtry* =5.

The simulation design for the second scenario was inspired by the set-up in [25, 31, 34]. The binary response *Y* was modeled by means of a logistic model:

$$P(Y = 1 | X = x) = \frac{e^{x^{T}\beta}}{1 + e^{x^{T}\beta}} $$

where the coefficients *β* were: *β*=(5,5,2,0,−5,−5,−2,0,0,0,0,0)^*T*^. The twelve predictors followed a multivariate normal distribution with mean vector *μ*=0 and covariance matrix *Σ*, with *σ*_*j*,*j*_=1 (all variables had unit variance), $\sigma _{j,j'} = 0.9\phantom {\dot {i}\!}$ for *j* ≠ *j* ^′^≤ 4 (the first four variables were block-correlated) and the other variables were independent with $\phantom {\dot {i}\!}\sigma _{j,j'} = 0$. The behavior of VIMs under predictor correlation could be studied with this model. As before, 120 observations were generated for 100 data sets.

The parameter settings for RFs were as follows. CART-RF was computed with bootstrap sampling without replacement, with *ntree* =500 as in [25], and two values for *mtry*: 3 (sqrt(*p*) the default value in [18]) and 12 (equal to *p*). GVIM, PVIM and IPM were computed for CART-RF. CIT-RF was computed with the settings suggested for the construction of an unbiased RF in [15], again with *ntree* =500 and *mtry* equal to 3 and 12. PVIM, CPVIM, and IPM were computed for CIT. varSelRF was used with the default parameters (*ntree* =5000). varSelMD was used with the default parameters (*ntree* =1000 and *mtry* =3) and also with *mtry* =12.

#### Multivariate responses: Scenarios 3 and 4

Again two scenarios were simulated. The design of the simulated data was inspired by the type of variable composition of the real problem with multivariate responses that would be analyzed. In this problem, responses were continuous and there were continuous and categorical predictors.

The configuration of the third and fourth scenarios were quite similar. Table 3 reports the predictor distributions, which were identical in both scenarios. Table 4 reports the response distributions, two continuous responses per scenario. Of the 7 predictors, only two were involved in the response simulation: the binary *X*_1_ and the continuous *X*_2_. However, in the fourth scenario *X*_2_ only participated in the response generation when *X*_1_=0. This arrangement was laid out in this way to analyze the ability of the methods to detect this situation. The rest of the predictors did not take part in the response generation, but *X*_5_ was very highly correlated with *X*_2_. In addition, the noise predictors *X*_6_ (continuous) and *X*_7_ (categorical) were created by randomly permuting the values of *X*_2_ and *X*_1_, respectively, as in [10]. The other irrelevant predictors, *X*_3_ and *X*_4_ were continuous with different distributions. As before, 120 observations in each scenario were generated for 100 data sets.

**Table 3:** Simulation design of predictors for Scenario 3 and 4

Variables	*X*_1_	*X*_2_	*X*_3_	*X*_4_	*X*_5_	*X*_6_	*X*_7_
Distribution	B(1,0.6)	N(3,1)	Unif(4,6)	N(5,1)	*X*_2_ + N(0,0.15)	P(*X*_2_)	P(*X*_1_)

The variables are sampled independently from the following distributions. B(1, *π*) stands for the Binomial distribution with *n* = 1, i.e the Bernoulli distribution, and probability *π*. N(*μ*,*σ*) stands for the normal distribution with mean *μ* and standard deviation *σ*. Unif(*a*,*b*) stands for the continuous uniform distribution on the interval [*a*,*b*]. P(*X*) stands for random permutation of the values generated in the variable *X*

**Table 4:** Simulation design of responses for Scenario 3 and 4

Variables	*Y*_1_|*X*_1_=0	*Y*_1_|*X*_1_=1	*Y*_2_|*X*_1_=0	*Y*_2_|*X*_1_=1
Scenario 3	2 + 2 ·*X*_2_ + N(0,0.1)	3 + 4 ·*X*_2_ + N(0,0.2)	2 + 3 ·*X*_2_ + N(0,0.15)	3 + 5 ·*X*_2_ + N(0,0.2)
Scenario 4	idem	4 + N(0,0.2)	idem	5 + N(0,0.2)

The variables are sampled independently from the following distributions. N(*μ*,*σ*) stands for the normal distribution with mean *μ* and standard deviation *σ*

With multivariate responses, only two VIMs could be computed. varSelMD was used with the default parameters (*ntree* =1000 and *mtry* =3) and also with *mtry* =7. IPM was computed for CIT-RF with the settings suggested for the construction of an unbiased RF in [15], with *ntree* =1000 and *m* *t* *r* *y*=7.

### Real data: Application to C-to-U conversion data and application to nutrigenomic study

Two well-known real data sets were analyzed. The first was a binary classification problem, where the predictors were of different types. In the second, the response was multivariate and the predictors were continuous and categorical, as in the first set.

The first data set was the *Arabidopsis thaliana, Brassica napus, and Oryza sativa* data from [14, 15], which can be downloaded from Additional file 2. It applies to C-to-U conversion data. RNA editing is the process whereby RNA is modified from the sequence of the corresponding DNA template [14, 15]. For example, cytidine-to-uridine (C-to-U) conversion is usual in plant mitochondria. Although the mechanisms of this conversion are not known, it seems that the neighboring nucleotides are important. Therefore, the data set is formed by 876 cases, each of them with the following recorded variables (one response and 43 predictors):

- Edit, with two values (edited or not edited at the site of interest). This is the binary response variable.
- The 40 nucleotides at positions –20 to 20 (named with those numbers), relative to the edited site, with 4 categories;
- the codon position, cp, which is categorical with 4 categories;
- the estimated folding energy, fe, which is continuous, and
- the difference in estimated folding energy (dfe) between pre-edited and edited sequences, which is continuous.

The second data set derives from a nutrigenomic study [16] and is available in the R package randomForestSRC [20] and Additional file 2. The study examines the effects of 5 dietary treatments on 21 liver lipids and 120 hepatic gene expressions in wild-type and PPAR-alpha deficient mice. Therefore, the continuous responses are the lipid expressions (21 variables), while the predictors are the continuous gene expressions (120 variables), the diet (categorical with 5 categories), and the genotype (categorical with 2 categories). The number of observations is 40. According to [16], in vivo studies were conducted under European Union guidelines for the use and care of laboratory animals and were approved by their institutional ethics committee.

## Results and discussion

### Scenario 1

**Fig. 1:**
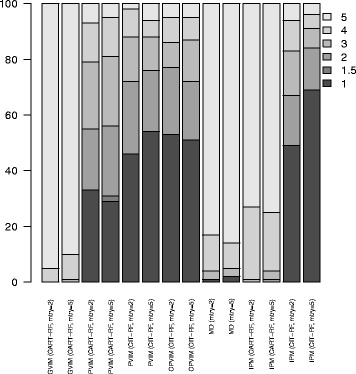
Ranking distribution of *X*_2_ for VIMs in Scenario 1. Barplots with the ranking distribution (in percentage) of *X*_2_. The darker the bar, the greater the importance of *X*_2_ for that method

**Fig. 2:**
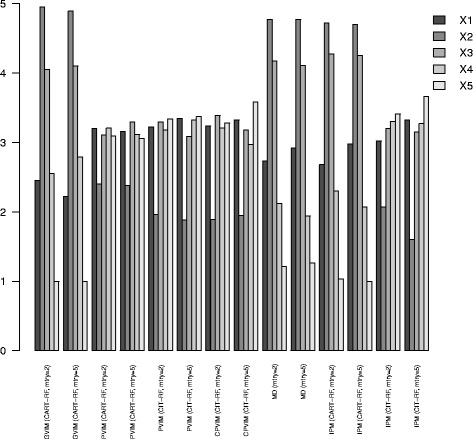
Average ranking of variables for VIMs in Scenario 1. Barplots with the average ranking of variables for VIMs. The lower the bar corresponding to *X*_2_, the greater the importance of *X*_2_ for that method

Figure 1 shows the ranking distribution of *X*_2_ for VIMs applied to Scenario 1. This information is also displayed in table form in Additional file 1: Table S1. The results for other sample sizes are shown in Additional file 1: Figures S1 and S2. In MD, the ranking according to minimal depth returned by the variable selection method varSelMD is shown. In theory, as *X*_2_ was the only relevant predictor, *X*_2_ should be in first place (the most important). The other uninformative variables should be in any place from 2nd to 5th, i.e. on average 3.5. The method which best identifies *X*_2_ as the most important predictor is IPM from CIT-RF with *mtry* =5, for which *X*_2_ was detected as the most important on 69% of occasions. The second best method is PVIM-CIT-RF with *mtry* =5, although it only identified *X*_2_ as the most important predictor on 54% of occasions. It is not surprising that the methods based on CART-RF do not obtain good results due to the nature of the problem, since there are different types of predictors and different

numbers of categories. In this situation, CIT provides a better alternative to CART, as is well explained in [15]. This statement is also corroborated by the results shown in Fig. 2, where the average rankings for each variable are shown. This information is also displayed in table form in Additional file 1: Table S2. The results for other sample sizes are shown in Additional file 1: Figures S3 and S4. Note that GVIM, MD and IPM from CART-RF selected *X*_2_ erroneously most times as the least important predictor, and *X*_5_, which is irrelevant, as the most important one. IPM from CIT-RF with *mtry* =5 was the method with the lowest average ranking for *X*_2_, i.e. that which gave the highest importance, in average terms, for *X*_2_. As regards the variable selection methods varSelRF and varSelMD, they have to be analyzed differently, as predictors are not ranked. The percentage of times that *X*_2_ belonged to the final selected model of varSelRF was 66%, despite selecting two variables on 82 occasions, three variables on 15 occasions and four variables on 3 occasions. Remember that only *X*_2_ was relevant. Note that IPM from CIT-RF with *mtry* =5 detected *X*_2_ as the most important predictor on 69% of occasions, and *X*_2_ was among the two most important predictors on 84% of occasions (much greater than 66%). The results for varSelMD were very poor with both *mtry* values. The method varSelMD with *mtry* =2 selected four predictors on 20% of occasions and five predictors, i.e. all the predictors, the remaining 80% of the times. It selects *X*_2_ on 80% of occasions, precisely when all the variables were chosen. In other words, it selected the four non-relevant variables and left *X*_2_ out of the model on 20% of occasions, and the remaining 80% of the times it did not make any selection, as it chose all the variables, including those which were irrelevant. The method varSelMD with *mtry* =5 selected all the predictors on 24% of occasions, four predictors 72% and three predictors 4%. *X*_2_ was among those selected on only 26% of occasions (when all the variable were selected on 24% of occasions).

IPM values are also easy to interpret, since they are positive and add one. The average IPM (from CIT-RF with *mtry* =5) values of cases in the 100 data sets for each variable were: 0.18 (*X*_1_), 0.31 (*X*_2_), 0.18 (*X*_3_), 0.17 (*X*_4_) and 0.16 (*X*_5_). So *X*_2_ was the most important, whereas it gave more or less the same importance to the other variables. An issue for further research is to determine from which threshold (maybe depending on the number of variables) a predictor can be considered irrelevant.

IPM can also be computed in class-specific terms, as PVIM-CART-RF. (They can also be computed casewise, but we omit those results in the interests of brevity). As an illustrative example, results from a data set are examined. In [11] we showed two problems for which the results of IPM by class were more consistent with that expected than those of PVIM-CART-RF by class, and this is also the case with the current problem. Table 5 shows the importance measures by group and globally. The IPM rankings seem to be more consistent at a glance than those for PVIM-CART-RF. For instance, the ranking by PVIM for class 1 gave *X*_1_ as the most important predictor, whereas *X*_1_ was the fourth (the penultimate) most important predictor for class 0. We computed Kendall’s coefficient *W* [35] to assess the concordance. Kendall’s coefficient W is an index of inter-rater reliability of ordinal data [36]. Kendall’s *W* ranges from 0 (no agreement) to 1 (complete agreement). Kendall’s W for the ranking of PVIM CART-RF (*mtry* =2) for class 0 and 1 was 0.5, whereas for IPM CIT-RF (*mtry* =5) it was 0.95. We repeated this procedure for each of the 100 data sets, and the average Kendall’s W were 0.71 and 0.96 for PVIM-CART-RF (*m* *t* *r* *y*=2) and IPM CIT-RF (*mtry* =5), respectively. Therefore, the agreement between the class rankings for IPM was very high. Note that in this case, the importance of predictors followed the same pattern for each response class as reflected by the IPM results, but it could be different in other cases. This has great potential in applied research, as explained in [32, 37]: for example, different predictors may be informative with different cancer subtypes.

**Table 5:** Analysis by class of a data set in Scenario 1

Measures	PVIM			IPM		
	0	1	G	0	1	G
*X*_1_	4	1	4	3	3	3
*X*_2_	2	3	2	1	1	1
*X*_3_	3	2	3	5	4	5
*X*_4_	1	4	1	2	2	2
*X*_5_	5	5	5	4	5	4

The first column is the name of the variables. The two following columns correspond to the PVIM ranking (CART-RF, *mtry* = 2) for each class, whereas the third column is the same but calculated globally (labeled as G). The last three columns contain the ranking of the IPM values (CIT-RF, *mtry* = 5) first by group and the last column computed globally (labeled as G)

### Scenario 2

According to the model generation, the most important variables were *X*_1_, *X*_2_, *X*_5_ and *X*_6_, which were equally important. The following variables in terms of importance were *X*_3_ and *X*_7_, which were also equally important. The other variables were irrelevant. However, there was a correlation pattern between variables *X*_1_, *X*_2_, *X*_3_ and *X*_4_, which were highly correlated, but they were uncorrelated to the other variables. Each VIM can be affected by this in different ways. Theoretically, if we rank the 12 variables by importance (from the most to the least important), the true ranking of each variable should be: 2.5, 2.5, 5.5, 9.5, 2.5, 2.5, 5.5, 9.5, 9.5, 9.5, 9.5, 9.5. Note that *X*_1_, *X*_2_, *X*_5_ and *X*_6_ should be in any of the first four positions, and 2.5 is the mean of 1, 2, 3 and 4. Analogously, *X*_3_ and *X*_7_ should be in 5th or 6th position, and 5.5 is the mean of these two values. Similarly, for the other variables, the mean of the 7th, 8th, 9th, 10th, 11th and 12th positions is 9.5.

**Fig. 3:**
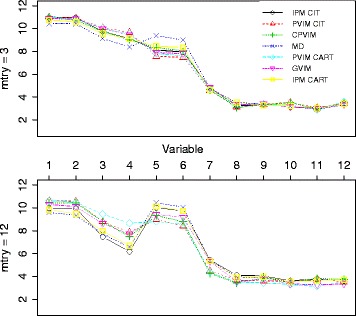
VIM rankings in Scenario 2. Average ranking in reverse order (a high number refers to high importance) for each VIM, for *mtry* =3 and *mtry* =12. The code of each VIM appears in the figure legend. As the *y* axis of the figure is flipped, 12 indicates a very important variable, whereas 1 a low important predictor. Theoretically, the true representation would be in this figure: 10.5, 10.5, 7.5, 3.5, 10.5, 10.5, 7.5, 3.5, 3.5, 3.5, 3.5, 3.5

Figure 3 shows the (flipped) average ranking (from the 100 data sets) for each method with *mtry* =3 and *m* *t* *r* *y*=12. The results for other sample sizes are shown in Additional file 1: Figures S5 and S6. As in [25], correlated predictors were given high importance with small *mtry* values, although *X*_3_ was not so important, even when *X*_4_ was irrelevant. Note that with *m* *t* *r* *y*=3, only MD gave higher importance (least ranking) to *X*_5_ and *X*_6_ than to the irrelevant *X*_4_. This is due to the high correlation with truly important variables *X*_1_ and *X*_2_. This effect is mitigated when *mtry* is increased. For *mtry* =12, the ranking profiles were more like the true one. The closest ranking profile to the true one was that given by IPM CIT-RF. The profiles of MD and IPM CART-RF were near to IPM CIT-RF, and all these methods are based on the tree structure. The IPM CIT-RF ranking for the most important (equally) variables was around 3 for all of them. For other methods there was more variation among the rankings of these four variables. For example, for CPVIM the average ranking for *X*_1_ and *X*_2_ was around 2.5, but it increased to around 4 for *X*_5_ and *X*_6_, despite being equally important. For the second equally important variables (*X*_3_ and *X*_7_), the IPM CIT-RF ranking gave a value of 5.5 for *X*_3_ (equal to the true one) and 7.5 for *X*_7_. The other methods gave more importance (lower ranking) to *X*_3_ and less importance to *X*_7_ (higher ranking), i.e. the other methods were further away from the true ranking. As regards the irrelevant variables, the IPM CIT-RF ranking gave a value of 6.8 for *X*_4_ and around 9 for the other uninformative variables. The *X*_4_ ranking for other methods was lower, i.e. they erroneously placed more importance on the uninformative variable *X*_4_. Therefore, IPM CIT-RF with *m* *t* *r* *y*=12 better identified the true pattern of importance.

The variable selection methods do not rank the variables, so the analysis can only be based on the selections. The distributions of the selections can be seen in Table 6. The number of variables selected by varSelRF varied from 2 to 8, although the most frequent numbers were 5 (on 31 occasions) and 6 (on 44 occasions). Note that the uninformative variable *X*_4_ was selected more times than the most important variables *X*_5_ and *X*_6_. As regards varSelMD, the results were good and were in line with MD. For *m* *t* *r* *y*=3, it selected 3 to 6 variables: four variables on 26 occasions, five variables on 51 occasions and six variables on 20 occasions. It detected the most important variables, although *X*_4_ was incorrectly selected 62% of the times, and *X*_7_ was only selected 3% of the times. The same happened for varSelMD with *m* *t* *r* *y*=12, although this time the number of *X*_4_ selections was lower (30%) and the number of *X*_7_ selections was higher (21%). The number of variables selected by varSelMD with *mtry* =12 ranged from 3 to 6, although it usually selected 4 (40%) or 5 (44%) variables. Remember that varSelRF takes into account the error rate for selecting the solution. This could be the reason why *X*_4_ is frequently selected by this method, because *X*_4_ is highly correlated with other variables in the model generation. This could also be the reason why the results for varSelRF in Scenario 1 were not as bad as for other methods based on CART-RF.

**Table 6:** Distribution (in percentage) of selections for variable selection methods in Scenario 2

Variable	*X*_1_	*X*_2_	*X*_3_	*X*_4_	*X*_5_	*X*_6_	*X*_7_	*X*_8_	*X*_9_	*X*_10_	*X*_11_	*X*_12_
varSelRF	98	97	90	87	82	63	6	2	2	2	1	1
varSelMD (*mtry* = 3)	97	97	74	62	85	70	3	0	0	0	0	0
varSelMD (*mtry* = 12)	79	76	48	30	96	92	21	2	5	1	5	3

#### IPM for a new case

**Fig. 4:**
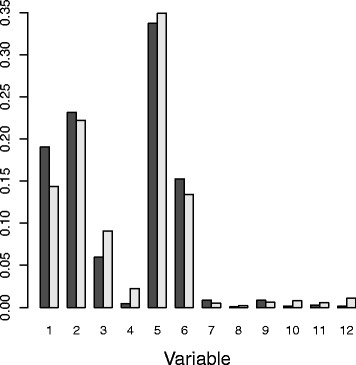
IPM for a new case in Scenario 2. IPM values with *mtry* =12 for CIT-RF (*dark grey*) and CART-RF (*light grey*)

IPM can be computed for a new case using either CIT-RF or CART-RF. Let us consider one of the data sets. A new artificial case with a value of zero in all the variables, i.e. the population mean of the generating model, can be built. According to the model construction, the importance pattern should be as discussed above. Figure 4 shows the IPM with *mtry* =12 for CIT-RF and CART-RF for this new case. Note that the other methods do not allow importance values to be computed for new cases. The four most important variables match the generating model. Variable *X*_3_ was the following most important. Variable *X*_7_ was as important as *X*_3_, but as before, its importance was underestimated. The importance of the other variables was negligible, although IPM CART-RF attached some importance to *X*_4_. Therefore, RF not only predicts the response of unlabeled samples (with unknown status), but also the importance of variables for each of those samples can be obtained with IPM. As discussed previously, local importance could reveal variables that are important for a subset of samples of the same class, which could be masked from global importance values [37]. This has potential and it is something to be explored further in the future, as only few studies have used local importances as yet [38, 39]. However, this should be approached with caution, as local importances could be noisier since they are based on smaller sample sizes.

### Application to C-to-U conversion data

First of all, let us analyze the VIMs. The composition of this data is similar to the data structure of Scenario 1 (predictors of different types), so only the results for the best methods in Scenario 1 are shown here. PVIM was computed with CIT-RF as in Scenario 1, with *n* *t* *r* *e* *e*=50 and *m* *t* *r* *y*=3 as in [15]. CPVIM could not be computed for this data set (not even if the threshold was changed) due to the high storage needs. But, as shown in [25], with increasing *mtry* values the unconditional importance resembled the behavior of the conditional importance, so PVIM-CIT-RF with *mtry* =43 was also considered. IPM-CIT-RF with *mtry* =43 was also computed.

**Fig. 5:**
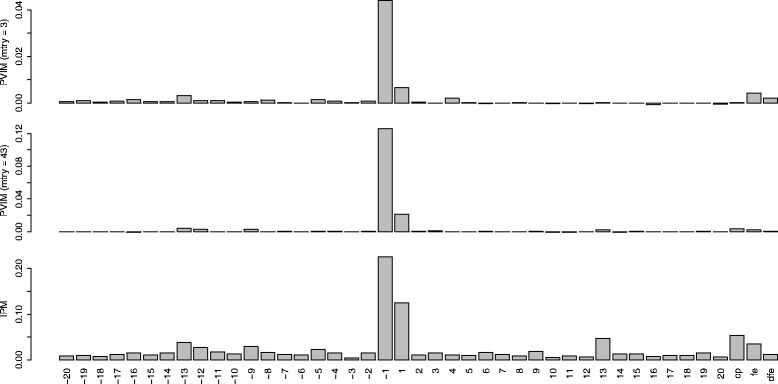
VIM barplots for the C-to-U conversion data. The first row corresponds to PVIM-CIT with *mtry* = 3, the second row to *mtry* = 43, while the bottom row to IPM-CIT-RF values with *mtry* = 43. In all cases, values are averaged over 100 replications. The variable names appear at bottom of the barplots

RFs are a randomized method. Therefore, the RF was computed 100 times from different seeds to gain stability in VIMs values. The VIM values from the 100 replications were averaged, and are displayed by barplots in Fig. 5. The results are similar to those in [15], although with some slight differences. As in [14, 15], position -1 was very important, followed by position 1, which was not detected in [14]. Note that GVIM, the method with the worst results in Scenario 1, was used in [14]. In [15], fe and dfe were somewhat important (only fe in [14]), but according to the results with PVIM (*mtry* =43) and IPM, cp (more than fe) and fe were somewhat important, but not dfe. Furthermore, according to IPM, there were also two somewhat important variables: positions -13 and 13.

In this real case, we do not know the true importance pattern of the variables. So, besides the variable importance study, let us also analyze the prediction accuracy in this data set. The same scheme as in [15] was considered. The original data were split into a training and test set with a size ratio of 2:1. This procedure was repeated 100 times. Each time the following operations were carried out. A RF-CART with bootstrap sampling without replacement, with *ntree* =50 and *mtry* =3 as in [15] was grown using the training set, and observations in the test set were predicted with this RF. The same procedure was performed with a CIT-RF with the settings suggested for the construction of an unbiased RF in [15], again with *ntree* =50 and *mtry* =3, as in [15]. These two procedures were used in [15]. On this occasion, the variable selection varSelRF was considered (varSelMD was not considered due to the poor results obtained in Scenario 1). An RF-CART was built as before, but using only the variables selected by varSelRF, and predictions in the test set were calculated. This is a kind of regularization, which attempts to erase noise (uninformative predictors) to improve the prediction. The same strategy was employed to exploit the best VIMs in Scenario 1. The idea was as follows. The same VIMs that appeared before in Fig. 5 were considered. The predictors were ranked according to these VIMs. The *m*=10 most important predictors were selected, and used to grow a CIT-RF with the settings suggested for the construction of an unbiased RF in [15], again with *n* *t* *r* *e* *e*=50 and *m* *t* *r* *y*=3. The test set was predicted by this RF. The value *m*=10 (a round number) was an intermediate value, not too small or too high, in view of the importance patterns displayed in Fig. 5 (there were not many important variables among the 43 predictors). The tuning parameter *m* should be further investigated, but the results are reported here without refining the value of *m*. The mean and standard deviation of the misclassification rates over the 100 runs appear in Table 7.

**Table 7:** Mean and standard deviation of misclassification rates in C-to-U conversion data

Method	RF-CART	RF-CIT	varSelRF	R-PVIM (*mtry* = 3)	R-PVIM (*mtry* = 43)	R- IPM
Mean	0.3051	0.2859	0.3054	0.2825	0.2793	0.2793
Std. deviation	0.0227	0.0250	0.0265	0.0219	0.0192	0.0208

Results produced by using the regularization procedure derived by VIMs are preceded by an R

The results obtained are similar those that appear in [15]. The greatest successes were achieved by methods conducted with CIT, as expected due to the data composition with predictors of different types. Furthermore, the regularization strategy using VIMs reported the best performance. In particular, the best results were achieved by using PVIM and IPM with CIT-RF and *m* *t* *r* *y*=43 to select the most important predictors before refitting the RF-CIT. In fact, both methods significantly (*p*-values of Student’s t-test for paired samples are well below 0.05) improved on the results given by RF-CART, CIT-RF and varSelRF.

### Scenarios 3 and 4

Tables 8 and 9 show the average ranking (from the 100 data sets) for each method. The results for other sample sizes are shown in Additional file 1: Tables S3, S4, S5 and S6. For scenario 3 and 4, variables *X*_1_ and *X*_2_ participated in the response generation, so both predictors were important. However, their importance level differs in each scenario. In scenario 4, *X*_2_ only intervened in the response generation when *X*_1_=0, so intuitively it should have had less importance than in scenario 3. As *X*_1_ and *X*_2_ participated in the response generation in a different way, and they are also variables of different types, it is difficult to judge their relative importance theoretically. In any case, *X*_1_ and *X*_2_ should rank in the first or second positions in scenario 3, while the other irrelevant variables should rank in 5th position (the mean of positions 3, 4, 5, 6, and 7).

**Table 8:** Average ranking of variables for VIMs in Scenario 3

Methods	*X*_1_	*X*_2_	*X*_3_	*X*_4_	*X*_5_	*X*_6_	*X*_7_
MD (*mtry* = 3)	1.36	2.04	4.85	4.99	2.60	5.16	7.00
MD (*mtry* = 7)	1.19	1.97	4.87	4.96	2.84	5.17	7.00
IPM (CIT-RF, *mtry* = 7)	2.00	1.00	5.41	5.40	3.40	5.43	5.37

**Table 9:** Average ranking of variables for VIMs in Scenario 4

Methods	*X*_1_	*X*_2_	*X*_3_	*X*_4_	*X*_5_	*X*_6_	*X*_7_
MD (*mtry* = 3)	1.00	2.28	5.07	4.97	2.74	4.94	7.00
MD (*mtry* = 7)	1.00	2.00	4.91	5.02	3.22	4.85	7.00
IPM (CIT-RF, *mtry* = 7)	1.00	2.06	4.59	4.71	6.11	4.75	4.78

In scenario 3, IPM considered *X*_2_ as the most important variable, followed by *X*_1_ in all the runs. The average IPM values were 32% for *X*_1_ and 68% for *X*_2_, and near zero for the other variables. The average rank for the other variables was around 5, except for *X*_5_ (the variable that was highly correlated with *X*_2_), with an average ranking of 3.4. Nevertheless, MD considered mostly *X*_1_ as the most important variable and in second position *X*_2_, although not in all the runs. For MD with *m* *t* *r* *y*=3, *X*_5_ was the most important on 8 occasions, and the second most important on 24 occasions. For MD with *m* *t* *r* *y*=7, *X*_5_ is the most important on 4 occasions, and the second most important on 8 occasions. Furthermore, MD always ranked *X*_7_ in last position (presumably because of its categorical nature), when it was no less important than the other uninformative variables.

In scenario 4, MD with *mtry* =7 considered *X*_1_ as the most important variable, followed by *X*_2_ in all the runs. On the other hand, MD with *mtry* =3 considered *X*_1_ as the most important in all the runs, as well as IPM. However, *X*_2_ was the second most important in all runs, except on 28 occasions for MD with *mtry* =3 and 6 occasions for IPM, where *X*_2_ was considered the third. The average IPM values were 40% for *X*_1_, 26% for *X*_2_ and around 7% for the other variables. However, as IPM is defined casewise, IPM can be also computed according to the group values of *X*_1_. Note that this kind of information supplied by IPM about importance in subgroups of data could only be available for MD if the RF was regrown with that subset of data. For samples with *X*_1_=0, the average IPM values were 47% for *X*_1_ and 53% for *X*_2_. Remember that when *X*_1_=0, the variable *X*_2_ intervened in the generation of the responses. For samples with *X*_1_=1 (*X*_2_ did not intervene in the generation of the responses), the average IPM values were 35% for *X*_1_, 7% for *X*_2_, 13% for *X*_3_, 13% for *X*_4_, 6% for *X*_5_, 13% for *X*_6_ and 12% for *X*_7_. Note that when *X*_1_=1, neither of the variables intervened in the model generation, so all the variables were equally unimportant. The selection frequency with CIT should be similar [15]. The sum of IPM of the two correlated variables *X*_2_ and *X*_5_ was 13%. Note also that this situation, where neither of the predictors is related with responses, is not expected (nor desirable) in practice.

### Application to a nutrigenomic study

Let us first analyze the VIMs. As the response is multivariate, only MD and IPM-CIT-RF can be computed. This problem is placed in a high-dimensional setting: it deals with large *p* (122) and small *n* (40). As explained in [10], as *p* increases, the tree becomes overwhelmed with variables, so trees will be too shallow. If we compute varSelMD and IPM-CIT-RF with *mtry* =122 and *n* *t* *r* *e* *e*=1000, only the variable diet is selected in both cases. This solution could be viewed as a ’degenerate’ solution. Then, the default *mtry* value in function rfsrc from R package randomForestSRC [20] is used. In this case *mtry* =*p*/3 (rounded up), i.e. *mtry* =41. A total of 34 variables were selected by varSelMD, diet being the least deep and genotype being the fourth least deep. However, except for diet, the depth values were not very different. To provide stability, this procedure was repeated 100 times with different seeds. A total of 44 variables were selected in some of the replicates. Half of these, 22 variables, were selected in all the replicates and 27 of them were selected on more than 75% of occasions. In particular, these were the following 27 predictors (the number of times they were selected over the 100 replicates is given in brackets): ACAT2 (100), ACBP (100), ACC2 (100), ACOTH (100), apoC3 (100), BSEP (89), CAR1 (100), CYP2c29 (100), CYP3A11 (100), CYP4A10 (100), CYP4A14 (100), diet (100), G6Pase (96), genotype (100), GK (77), GSTpi2 (100), HPNCL (100), Lpin (100), Lpin1 (100), Lpin2 (97), Ntcp (100), PLTP (100), PMDCI (100), S14 (100), SPI1.1 (100), SR.BI (99), and THIOL (100).

**Fig. 6:**
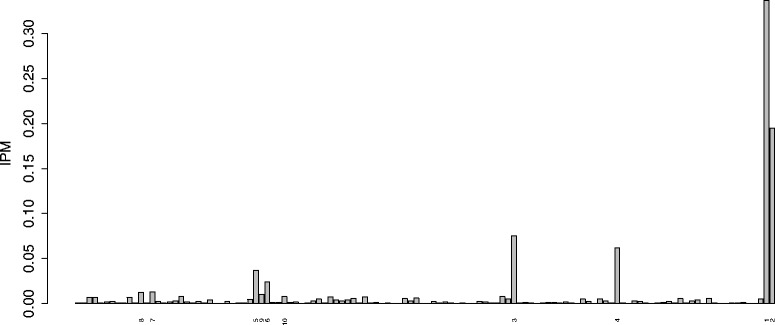
Barplot of averaged IPM values for nutrigenomic study. The ranking of the 10 predictors with the largest IPM values appears at the bottom. Their names can be found in Table 10

For the 100 replicates of IPM-CIT-RF with *m* *t* *r* *y*=41, the ranking of the first variables was very stable: diet was the first in all replicates, and genotype the second. The average ranking of the first ten ranked predictors together with the standard deviation for the 100 replicates can be seen in Table 10. Although not suggested by the varSelMD results, the IPM values indicate that four variables accounted for most relevance (nearly 70%). In particular, these are the averaged IPM values for the four variables in brackets: diet (33.7%), genotype (19.5%), PMDCI (7.5%) and THIOL (6.2%). The barplot of these IPM values can be seen in Fig. 6. The seventh and eight most important predictors according to IPM, BIEN and AOX were not selected in any of the replications of varSelMD.

**Table 10:** Average ranking of the first 10 ranked variables in the nutrigenomic study for IPM (SD in brackets)

diet	genotype	PMDCI	THIOL	CYP3A11	CYP4A14	BIEN	AOX	CYP4A10	FAS
1 (0)	2 (0)	3.1 (0.3)	3.9 (0.4)	5 (0.2)	6.1 (0.8)	9.1 (3.6)	9.1 (3)	12.4 (5.2)	17 (8.6)

**Fig. 7:**
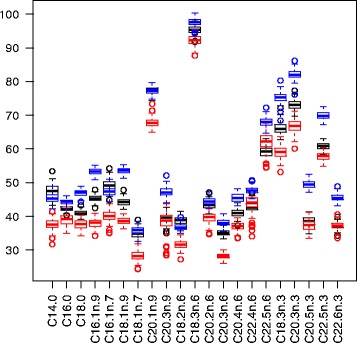
Boxplots of standardized prediction errors for nutrigenomic study. Distributions of standardized prediction errors for each variable, for methods rfsrc (in *blue*), varSelMD (in *black*) and IPM (in *red*) from 100 replications

Let us analyze the prediction performance. Prediction error was calculated using OOB data. As the responses were continuous, performance was measured in terms of mean-squared-error. The prediction error for each of the 21 responses was standardized (dividing by the variance as in the R package randomForestSRC [20]) for proper comparison. The prediction error using the function rfsrc from R package randomForestSRC with the default values was computed, which is referred to as rfsrc. varSelMD was applied as before, and the function rfsrc was again used for prediction afterwards, but the selected variables were used as input instead all the variables. This procedure is referred as varSelMD. Finally, instead of varSelMD, IPM-CIT-RF was applied as before, and the 10 most important variables were selected, for prediction. This procedure was referred as IPM. The standardized errors for each response were averaged over 100 independent experiments, and their results are summarized in Table 11 and displayed in Fig. 7. The lower prediction errors from IPM can be clearly observed. Furthermore, by pooling the samples for each variable and using a Student’s t-test for paired samples with *α*=0.05, IPM significantly improves varSelMD, which in turn improves rfsrc.

**Table 11:** Mean and standard deviation of standardized prediction errors in the nutrigenomic study

Method	rfsrc	varSelMD	IPM
Mean	54.81	50.13	45.61
Std. deviation	16.35	15.72	15.71

### What are the advantages and limitations of IPM?

One of the advantages is the out-performance of IPM in the previous comparisons. In addition, its case-wise constitution should also be highlighted. IPM can be defined globally, for each class (for RF-classification) and locally, as well as for new cases (even if we do not know their true response). Only PVIM can also be computed locally, by class and globally, but except for IPM, none of the other methods are able to estimate a variable’s influence in a new case without knowing its true response. Furthermore, IPM can be computed for subsets of data, with no need to regrow the forest for those subsets.

Furthermore, IPM is independent of any prediction error, like MD. This is very advantageous as it is not always known how the prediction error can be clearly measured [33], and VIMs (rankings of important variables) can be different if different prediction error measures are employed. Furthermore, as IPM is only based on the tree structures, it can be applied to all kind of forests, regardless of the outcome, from RF-classification to Multivariate RFs. [33] indicates another possible advantage of MD due to the fact that it is not linked to any prediction error, which is also the case of IPM. Although RFs are excellent in prediction for high dimensions, prediction performance breaks down when the number of noisy predictors increases, as it overwhelms the trees. As a consequence, it is difficult to select variables effectively, and methods that are based on prediction error, such as PVIM, may be more susceptible to these effects than methods based on tree structure. As long as a predictor *v* repeatedly splits across the forest, MD or IPM have a good chance of identifying *v*, even in the presence of many noisy predictors.

As regards computational complexity, IPM basically depends on exploring the trees for each observation, so the most computational burden part is growing the trees. In addition, IPM is conceptually simple and its interpretation is very accessible for everyone as it is expressed in percentages.

The influence of sample size on the performance of IPM is investigated in the simulated scenarios. Results are shown for sample sizes of *n*=50 and *n*=500 in Additional file 1. As indicated by [40], the data sets are usually high dimensional, i. e. with a small sample size relative to the dimension, and RFs with the largest trees are optimal in such studies. However, this is not the case with *n*=500, where there are few variables with a high number of observation. In such situations, it is desirable to have the terminal node size go up with the sample size [40]. In those cases, the maximum depth (*maxdepth*) of the trees in RFs may regulate overfitting [41]. As IPM results are based only on the tree structure, it is fundamental to grow trees that are not overfit, otherwise noise is introduced, which can distort the results.

The drawbacks of IPM are common to any other rank-based VIM, in the sense that caution is needed when interpreting any linear ranking because it is possible that multiple sets of weak predictive variables may be jointly predictive.

## Conclusion

A new VIM for RFs, IPM, has been introduced and assessed in different scenarios, within both simulated and real frameworks. IPM can be used in place of (or in addition to) other VIMs. The advantages and limitations of IPM have been highlighted in the previous Section.

There also some questions that deserve further research, such as the choice of *mtry* or *maxdepth* (for a high *n*) in RFs for IPM computation. As the objective of IPM is not prediction, but to indicate the contribution of variables to prediction, high *mtry*s with CIT-RF have given very good performance in the simulation studies carried out. However, when *p* >*n*, *mtry* should be reduced. Besides the qualitative information provided by IPM for understanding problems, if we want to use that information for predicting, we have to select a threshold for selecting the variables for regrowing the RF. In the problems, a round fixed number of the 10 variables with the highest IPM values was selected for predicting purposes, with promising results. An open question would be to explore the selection of this number and its relationship with the distribution of IPM values (possibly selecting variables with IPM values above a certain threshold), together with the number of observations and predictors. A detailed study should be made with scenarios covering *p* >*n* cases, such as scenarios with a few relevant variables and many irrelevant variables and scenarios with many slightly relevant variables. Another open question it is to try to perform a theoretical study of IPM, as in [10] for MD. Note that, according to [42], it seems very difficult to carry out a detailed theoretical study of PVIM, but IPM is not a randomization procedure like PVIM.

## Additional files

Additional file 1 Supplementary file. This file shows the results of VIMs in the simulated scenarios for sample sizes of *n*=50 and *n*=500. **Figure S1**: Ranking distribution (in percentage) of *X*_2_ for VIMs in Scenario 1 with *n* = 50. **Figure S2**: Ranking distribution (in percentage) of *X*_2_ for VIMs in Scenario 1 with *n*=500. **Figure S3**: Average ranking of variables for VIMs in Scenario 1 with *n*=50. **Figure S4**: Average ranking of variables for VIMs in Scenario 1 with *n*=500. **Table S1**: Ranking distribution (in percentage) of *X*_2_ for VIMs in Scenario 1 with *n* = 120. **Table S2**: Average ranking of variables for VIMs in Scenario 1 with *n*=120. **Figure S5**: Average ranking for each VIM in Scenario 2, for *mtry* =3 and *mtry* =12, with *n*=50. **Figure S6**: Average ranking for each VIM in Scenario 2, for *mtry* =3 and *mtry* =12, with *n*=500. **Table S3**: Average ranking of variables for VIMs in Scenario 3, with *n*=50. **Table S4**: Average ranking of variables for VIMs in Scenario 3, with *n*=500. **Table S5**: Average ranking of variables for VIMs in Scenario 4, with *n*=50. **Table S6**: Average ranking of variables for VIMs in Scenario 4, with *n*=500. (PDF 166 kb)

Additional file 2 R source code. This is a compressed (.zip) file with data and R codes for reproducing the results. There is a file called Readme.txt that explains the contents of six files. Two files contain the two real data sets. The R code for computing IPM for RF-CART and CIT-RF is available in one file. The R code for each of the simulations is available in another file, while the other two files contain the R codes for each application to real data. (ZIP 43 kb)

## Abbreviations

CPVIM: Conditional permutation VIM
CART: Classification and Regression Trees
CIT: Conditional Inference Trees
CART-RF: Random forest based on CART
CIT-RF: Random forest based on CIT
Cytidine-to-Uridine (C-to-U); GVIM: Gini VIM
IPM: Intervention in prediction measure
MD: Minimal depth
OOB: Out-of-bag
PVIM: Permutation VIM
PVIM-CIT-RF: PVIM derived from CIT-RF
PVIM-CART-RF: PVIM derived from CART-RF
RF: Random forest
Residual Sum of Squares (RSS); varSelRF: Variable selection procedure proposed by [26]
varSelMD: Variable selection procedure using MD proposed by [10]
VIM: Variable importance measure
