# Supplementary material for: Intervention in prediction measure: a new approach to assessing variable importance for random forests
Source: BMC Bioinformatics. 2017 May 2;18:230. doi: 10.1186/s12859-017-1650-8 (PMC5414143; doi:10.1186/s12859-017-1650-8)
Supplement: Supplementary file 1 — Supplementary file. This file shows the results of VIMs in the simulated scenarios for sample sizes of n=50 and n=500. Figure S1: Ranking distribution (in percentage) of X 2 for VIMs in Scenario 1 with n = 50. Figure S2: Ranking distribution (in percentage) of X 2 for VIMs in Scenario 1 with n=500. Figure S3: Average ranking of variables for VIMs in Scenario 1 with n=50. Figure S4: Average ranking of variables for VIMs in Scenario 1 with n=500. Table S1: Ranking distribution (in percentage) of X 2 for VIMs in Scenario 1 with n = 120. Table S2: Average ranking of variables for VIMs in Scenario 1 with n=120. Figure S5: Average ranking for each VIM in Scenario 2, for mtry =3 and mtry =12, with n=50. Figure S6: Average ranking for each VIM in Scenario 2, for mtry =3 and mtry =12, with n=500. Table S3: Average ranking of variables for VIMs in Scenario 3, with n=50. Table S4: Average ranking of variables for VIMs in Scenario 3, with n=500. Table S5: Average ranking of variables for VIMs in Scenario 4, with n=50. Table S6: Average ranking of variables for VIMs in Scenario 4, with n=500. (PDF 166 kb) [file 12859_2017_1650_MOESM1_ESM.pdf]

Supplementary material for "Intervention in prediction measure: a new approach to assessing variable importance for random forests"

Irene Epifanio
Dept. Matemàtiques and IMAC
Universitat Jaume I
Castelló, 12071
Spain

April 7, 2017

Abstract

The influence of sample size on the performance of IPM is investigated in the simulated scenarios. Results are shown for sample sizes of $n = 50$ and $n = 500$. The figures and tables appearing in the manuscript for $n = 120$ are replicated for these sample sizes.

1 Scenario 1

Figures S1 and S2 are analogous to Figure 1 in the manuscript, while Figures S3 and S4 correspond to Figure 2 in the manuscript with sample sizes $n = 50$ and $n = 500$, respectively. With $n = 50$, results are similar to those in the manuscript with $n = 120$. With $n = 500$, there are few variables with a high number of observation. In such situations, it is desirable to have the terminal node size go up with the sample size [3]. In the previous figures, this was not taken into consideration and IPM results have been affected, as they are based only on the tree structure, and not on performance. In

those cases, the depth of the trees in random forests may regulate overfitting [4]. If the maximum depth (*maxdepth*) of the trees are restricted to 3 (this parameter has not been tuned), for example, results for IPM change for the better radically. The average ranking of variables for IPM (CIT-RF, $mtry = 5$, $maxdepth = 3$) in the case of $n = 500$ is: 3.28 ($X1$), 1.01 (X_2), 3.63 (X_3), 3.47 (X_4) and 3.61 (X_5), i.e. X_2 ranks first on 99% of occasions, and second on 1% of occasions. Therefore, the ranking configuration is nearly perfect. For comparison, Table S1 shows the ranking distribution of X_2 for VIMs applied to Scenario 1 with $n = 120$, as in the manuscript, whereas the average rankings for each variable are shown in Table S2.

Table S1: Ranking distribution (in percentage) of X_2 for VIMs in Scenario 1 with $n = 120$. The most frequent position for each method is marked in bold font. Note that X_2 should rank ideally first in 100% of occasions.

Methods	1	1.5	2	3	4	5
GVIM (CART-RF, $mtry = 2$)					5	**95**
GVIM (CART-RF, $mtry = 5$)				1	9	**90**
PVIM (CART-RF, $mtry = 2$)	**33**		22	24	14	7
PVIM (CART-RF, $mtry = 5$)	**29**	2	25	25	14	5
PVIM (CIT-RF, $mtry = 2$)	**46**		26	16	10	2
PVIM (CIT-RF, $mtry = 5$)	**54**		22	12	6	6
CPVIM (CIT-RF, $mtry = 2$)	**53**		24	9	9	5
CPVIM (CIT-RF, $mtry = 5$)	**51**		21	15	8	5
MD ($mtry = 2$)	1			3	13	**83**
MD ($mtry = 5$)	2			3	9	**86**
IPM (CART-RF, $mtry = 2$)				1	26	**73**
IPM (CART-RF, $mtry = 5$)			1	3	21	**75**
IPM (CIT-RF, $mtry = 2$)	**49**		18	16	11	6
IPM (CIT-RF, $mtry = 5$)	**69**		15	7	5	4

2 Scenario 2

Figures S5 and S6 show the average ranking (from the 100 data sets) for each method with $mtry = 3$ and $mtry = 12$, and $n = 50$ and $n = 500$, respectively. They are analogous to Figure 3 in the manuscript with $n = 120$. With $n = 50$ and $mtry = 3$ results of all methods are quite similar among them.

Table S2: Average ranking of variables for VIMs in Scenario 1 with $n = 120$. The most important variable (lowest ranking) for each method is marked in bold font. Ideally, X_2 should rank 1, and 3.5 the rest of variables.

Methods	X_1	X_2	X_3	X_4	X_5
GVIM (CART-RF, $mtry = 2$)	2.45	4.95	4.05	2.55	**1.00**
GVIM (CART-RF, $mtry = 5$)	2.22	4.89	4.10	2.79	**1.00**
PVIM (CART-RF, $mtry = 2$)	3.20	**2.40**	3.11	3.21	3.09
PVIM (CART-RF, $mtry = 5$)	3.16	**2.38**	3.29	3.12	3.06
PVIM (CIT-RF, $mtry = 2$)	3.23	**1.96**	3.30	3.18	3.34
PVIM (CIT-RF, $mtry = 5$)	3.35	**1.88**	3.09	3.32	3.37
CPVIM (CIT-RF, $mtry = 2$)	3.24	**1.89**	3.39	3.21	3.28
CPVIM (CIT-RF, $mtry = 5$)	3.32	**1.95**	3.18	2.97	3.58
MD ($mtry = 2$)	2.73	4.77	4.17	2.12	**1.21**
MD ($mtry = 5$)	2.92	4.77	4.11	1.94	**1.26**
IPM (CART-RF, $mtry = 2$)	2.68	4.72	4.27	2.30	**1.03**
IPM (CART-RF, $mtry = 5$)	2.98	4.70	4.25	2.07	**1.00**
IPM (CIT-RF, $mtry = 2$)	3.02	**2.07**	3.20	3.30	3.41
IPM (CIT-RF, $mtry = 5$)	3.32	**1.60**	3.15	3.27	3.66

With $mtry = 12$, the smaller sample size affects the methods differently. PVIM-CIT-RF and CPVIM provide less importance to X_5 and X_6 than to the irrelevant variable X_4. MD considers X_5 and X_6 more important than X_4, but the importance of X_1 and X_2 is not as high as expected, and it is too similar to the importance given to (the less important) X_3 and the irrelevant X_4. IPM-CIT-RF shows a ranking pattern in the middle between these two situations, the one represented by PVIM-CIT-RF and CPVIM, and the one represented by MD.

As regards the results with $n = 500$, on the one hand the higher sample size affects the behavior of the methods in three different ways with $mtry = 3$. Results with PVIM-CIT-RF, PVIM-CART-RF and GVIM are the less successful because give more or less the same importance to the irrelevant variable X_4 as the important predictors X_5 and X_6. The opposite behavior is found for CPVIM, which is the method with the biggest difference in importance between X_4 and the group formed by X_5 and X_6. However, CPVIM gives less importance to the relevant predictors X_1 and X_2, when they are as important as X_5 and X_6. IPM (CIT-RF and CART-RF) and MD show a similar profile as CPVIM, but they give more importance to X_1 and

X_2 than the one given to CPVIM, and less importance to X_5 and X_6 than the one given by CPVIM. On the other hand, with $mtry = 12$ the methods show a similar ranking pattern among them, but the methods that give the most similar ranking to the theoretical one are IPM with CIT-RF and CART-RF. The dissimilarity is computed as the sum of the differences in absolute value between the average ranking of each method and the theoretical one.

3 Scenarios 3 and 4

Tables S3, S4, S5 and S6 show the average ranking (from the 100 data sets) for each method in Scenarios 3 and 4 with $n = 50$ and $n = 500$. They are homologue to Tables 8 and 9 in the manuscript with $n = 120$.

Table S3: Average ranking of variables for VIMs in Scenario 3, with n =50.

Methods	X_1	X_2	X_3	X_4	X_5	X_6	X_7
MD ($mtry = 3$)	1.84	1.79	5.05	4.93	2.37	5.06	6.96
MD ($mtry = 7$)	1.36	1.98	4.97	5.00	2.66	5.04	6.99
IPM (CIT-RF, $mtry = 7$)	2.21	1.02	5.44	5.42	3.07	5.42	5.44

Table S4: Average ranking of variables for VIMs in Scenario 3, with n =500.

Methods	X_1	X_2	X_3	X_4	X_5	X_6	X_7
MD ($mtry = 3$)	1.00	2.01	5.06	4.94	2.99	5.00	7.00
MD ($mtry = 7$)	1.00	2.00	4.89	5.06	3.00	5.05	7.00
IPM (CIT-RF, $mtry = 7$)	2.00	1.00	5.18	5.20	4.21	5.28	5.14

Table S5: Average ranking of variables for VIMs in Scenario 4, with $n = 50$.

Methods	X_1	X_2	X_3	X_4	X_5	X_6	X_7
MD ($mtry = 3$)	1.00	2.71	4.75	4.79	2.93	4.82	7.00
MD ($mtry = 7$)	1.00	2.46	4.60	4.74	3.14	5.07	6.99
IPM (CIT-RF, $mtry = 7$)	1.00	4.41	4.26	4.19	4.82	4.52	4.82
IPM (CIT-RF, $mtry = 7$, all samples)	1.03	3.64	4.43	4.52	4.83	4.84	4.72

Table S6: Average ranking of variables for VIMs in Scenario 4, with $n = 500$.

Methods	X_1	X_2	X_3	X_4	X_5	X_6	X_7
MD ($mtry$ = 3)	1.00	2.10	5.03	4.94	2.90	5.03	7.00
MD ($mtry$ = 7)	1.00	2.00	4.38	4.46	4.46	4.70	7.00
IPM (CIT-RF, $mtry$ = 7)	2.01	1.00	4.15	4.29	6.28	4.24	6.03
IPM (CIT-RF, $mtry$ = 7, $maxdepth$ = 3)	1.19	1.81	4.62	4.63	5.90	4.74	5.11

For Scenario 3, X_1 or X_2 are in third position in 50% of occasions with n = 50 and $mtry$ = 3 with MD, their results are more affected to worse by a smaller sample size than the results of the other methods. X_5 (related with X_2) is given lower ranking in all methods than when $n = 120$ was considered, but the same patterns in Table 8 are observed in general. However, with n = 500, results for IPM are more similar to those theoretically expected (X_1 and X_2 in first position, while the rest of variables are irrelevant and should rank in 5th position). For MD with $n = 500$, the pattern observed with $n = 120$ in Table 8 is now more evident. Results with MD shows a bias on the irrelevant categorical predictor X_7, which is always ranked in 7th position, and also on X_5 (irrelevant but related with X_2), which is always ranked in 3rd position. The other irrelevant variables X_3, X_4 and X_6 rank in 5th position.

In Scenario 4 with $n = 50$, results are affected for the small sample size and the special configuration. Remember that variable X_2 is irrelevant when X_1 = 1, which is the most frequent value (60%). In other words, it is expected that X_2 intervenes in the generation of approximately only 20 (50 × 0.4) samples. With MD, the rank of X_2 rises up and it is closer to the rank given to X_5. The same ranking pattern as that of the case n = 120 is observed for the rest of variables, included the bias for X_7. For IPM, the rank of X_2 rises a lot with n = 50. X_2 ranks in second position in 22% of occasions, while X_2 ranks from third to seventh position around 15% of occasions for each position. Note that when n = 50, the size of the OOB sample is around 18 (50 × (1-0.632)), so only around 7 (18 × 0.4) samples will have a value of X_1 = 0 and X_2 will participate in the generation of the responses. The size sample of the in-bag observations, which build the trees, with X_1 = 0 is approximately 13 (50 × 0.632 × 0.4). Therefore, we are estimating IPM with a very small sample, and a small sample size is a source of variance [1]. For solving this issue and increasing the sample size, trees have been computed with all available observations ($fraction$ = 0.99

has been considered in the function $cforest$ of the R package party [2]) as IPM is not used for prediction. Then, all observations have been used for estimating IPM. Results of this configuration appear in the last row of Table S5, which gives an average ranking of 3.64 for X_2. The average ranking for irrelevant variables is around 4.5. However, this global information can be easily desegregated by groups with IPM, supplying interesting information. The average IPM values were 54% for X_1, 15% for X_2 and around 6% for the other variables. For samples with $X_1 = 0$, the average IPM values were 75% for X_1 and 25% for X_2, and null for the rest of variables. Therefore, IPM discards the irrelevant variables.

Results for VIMs with $n = 500$ in Scenario 4 are similar to the manuscript with $n = 120$, except for IPM, for the reason explained in Section 1. The results for IPM (CIT-RF, $mtry = 7$, $maxdepth = 3$) are incorporated into Table S6. When the depths of trees are limited for avoiding overfitting in the high sample size setting, results of IPM are again very good. In fact, it is very reasonable that X_1 ranks first and X_2 second in 81% of occasions, and X_2 ranks first and X_1 second in 19% of occasions, according to the structure of Scenario 4, since X_1 is only important for some part of the sample, and both X_1 and X_2 are important for the other part of the sample.

References

[1] T. Hastie, R. Tibshirani, and J. Friedman. *The Elements of Statistical Learning. Data mining, inference and prediction.* 2nd ed., Springer-Verlag, New York, 2009.

[2] T. Hothorn, K. Hornik, C. Strobl, and A. Zeileis. *A Laboratory for Recursive Partytioning*, 2016. R package version 1.0.25.

[3] Y. Lin and Y. Jeon. Random forests and adaptive nearest neighbors. *Journal of the American Statistical Association*, 101(474):578–590, 2006.

[4] C. Strobl, J. Malley, and G. Tutz. An introduction to recursive partitioning: Rationale, application, and characteristics of classification and regression trees, bagging, and random forests. *Psychological Methods*, 14(4):323–348, 2009.

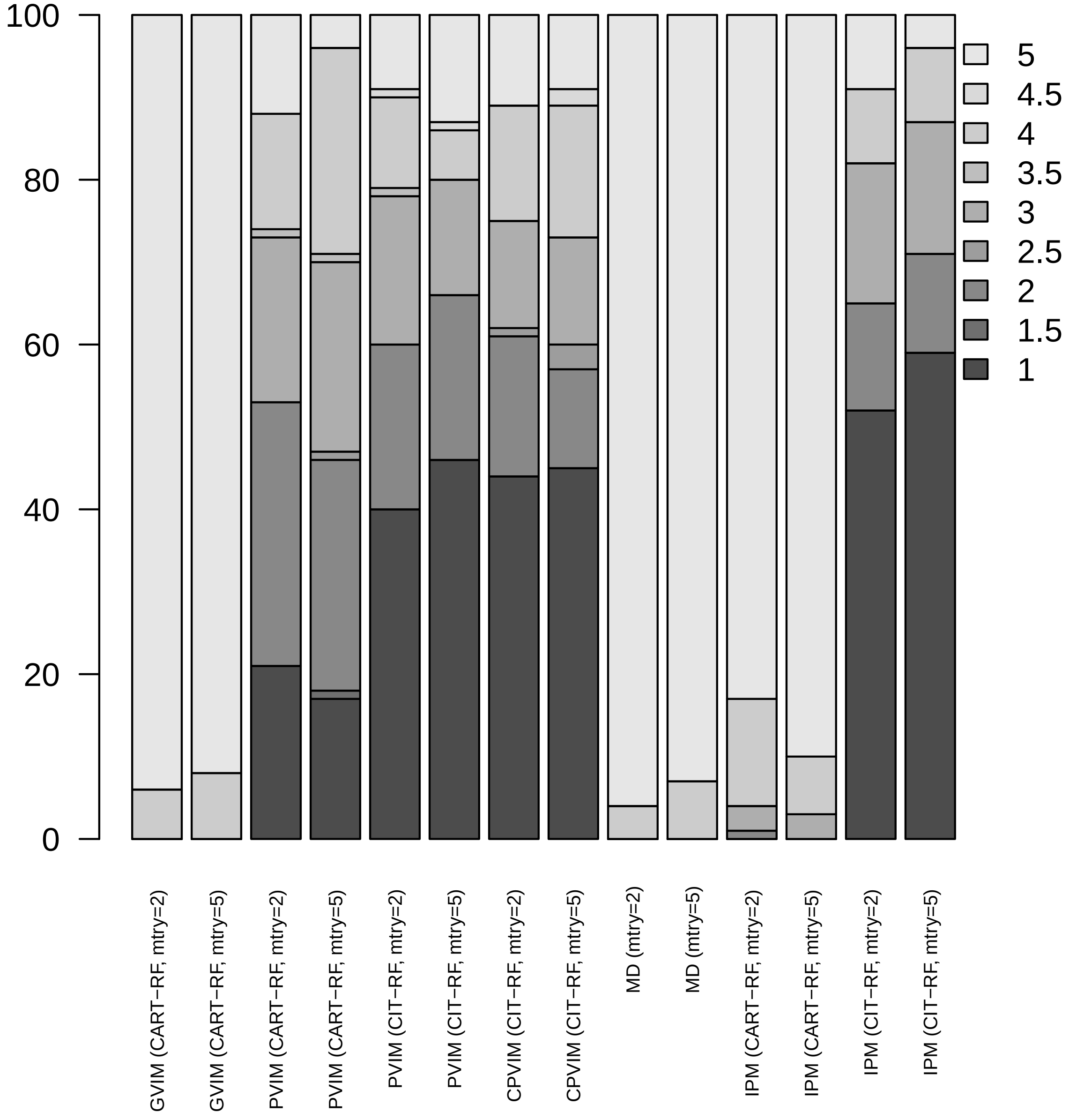

Figure S1: Ranking distribution (in percentage) of X_2 for VIMs in Scenario 1 with $n = 50$.

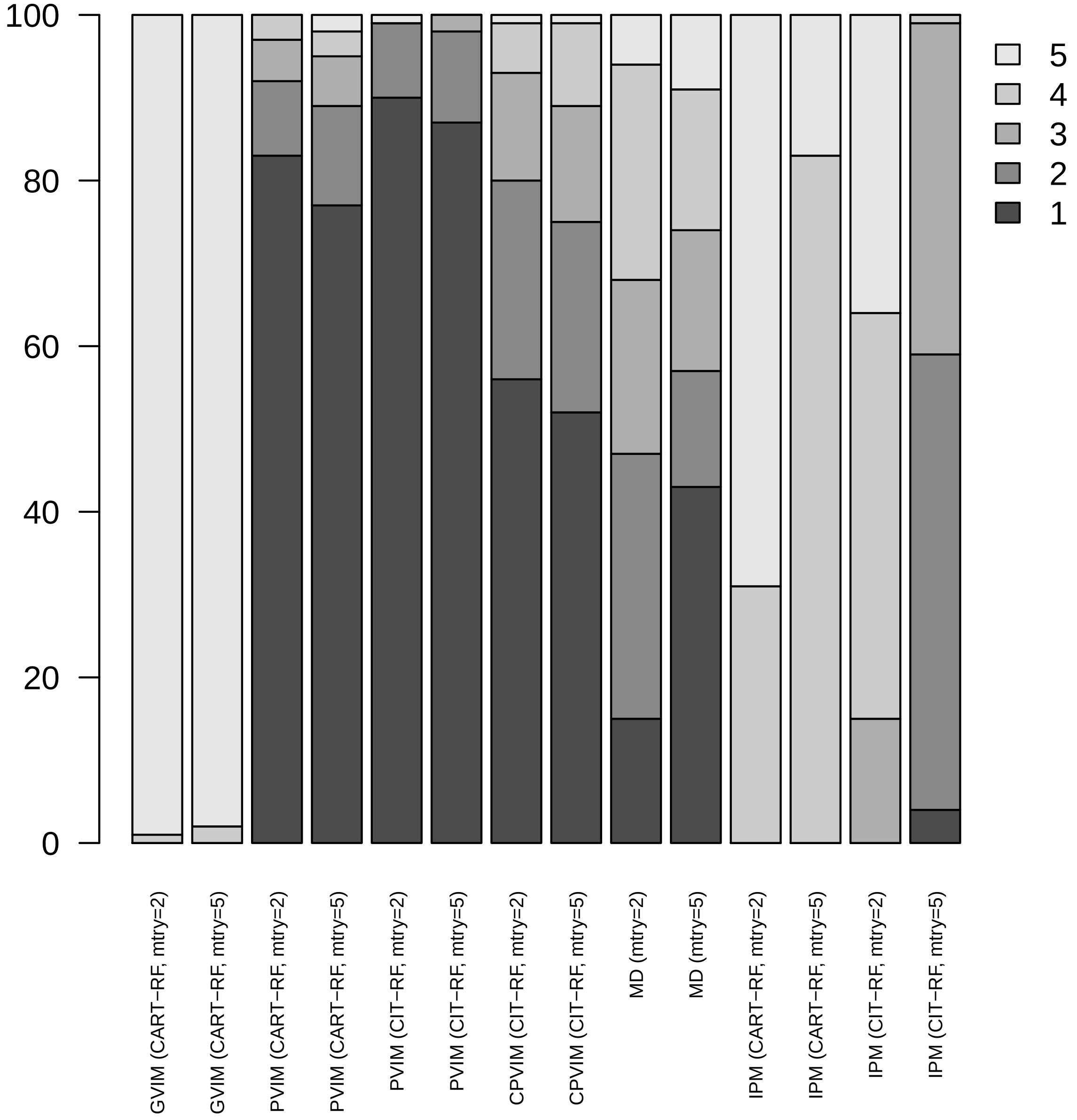

Figure S2: Ranking distribution (in percentage) of X_2 for VIMs in Scenario 1 with $n = 500$.

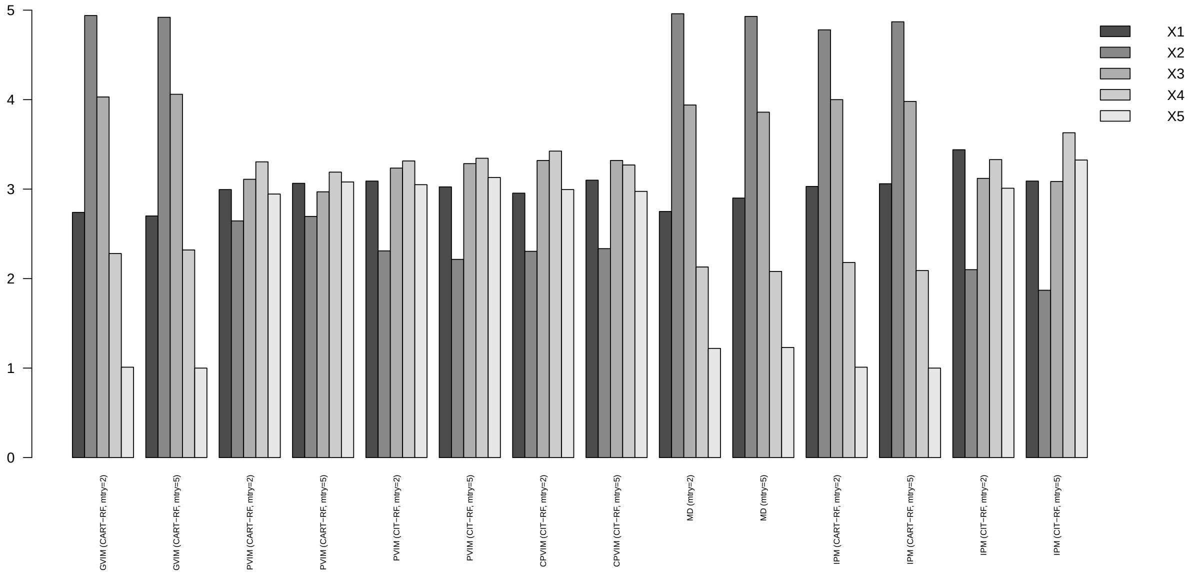

Figure S3: Average ranking of variables for VIMs in Scenario 1 with $n = 50$.

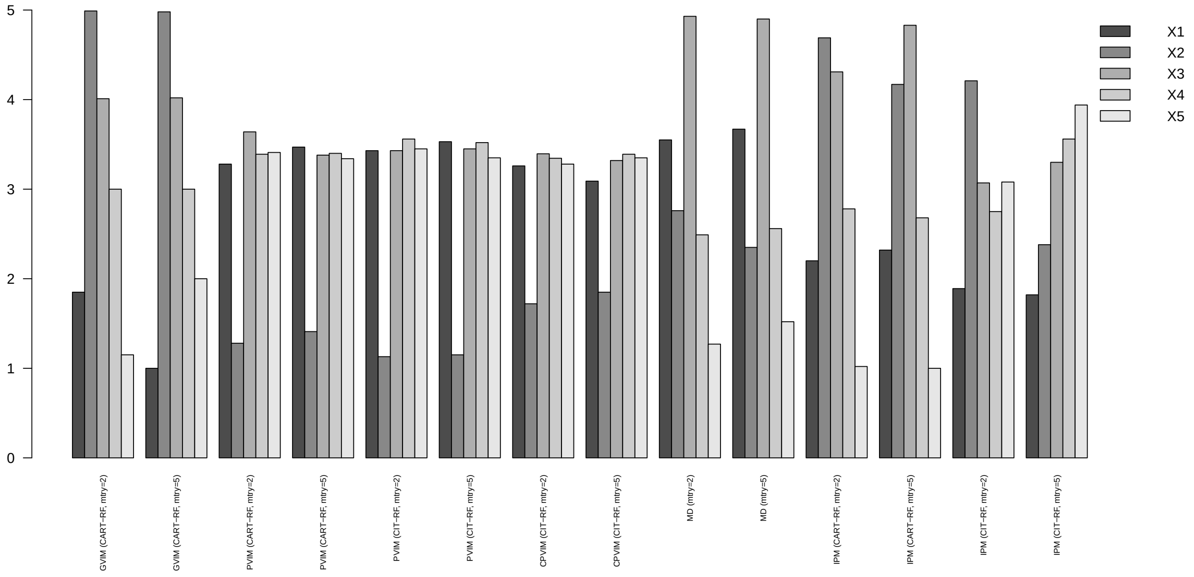

Figure S4: Average ranking of variables for VIMs in Scenario 1 with $n = 500$.

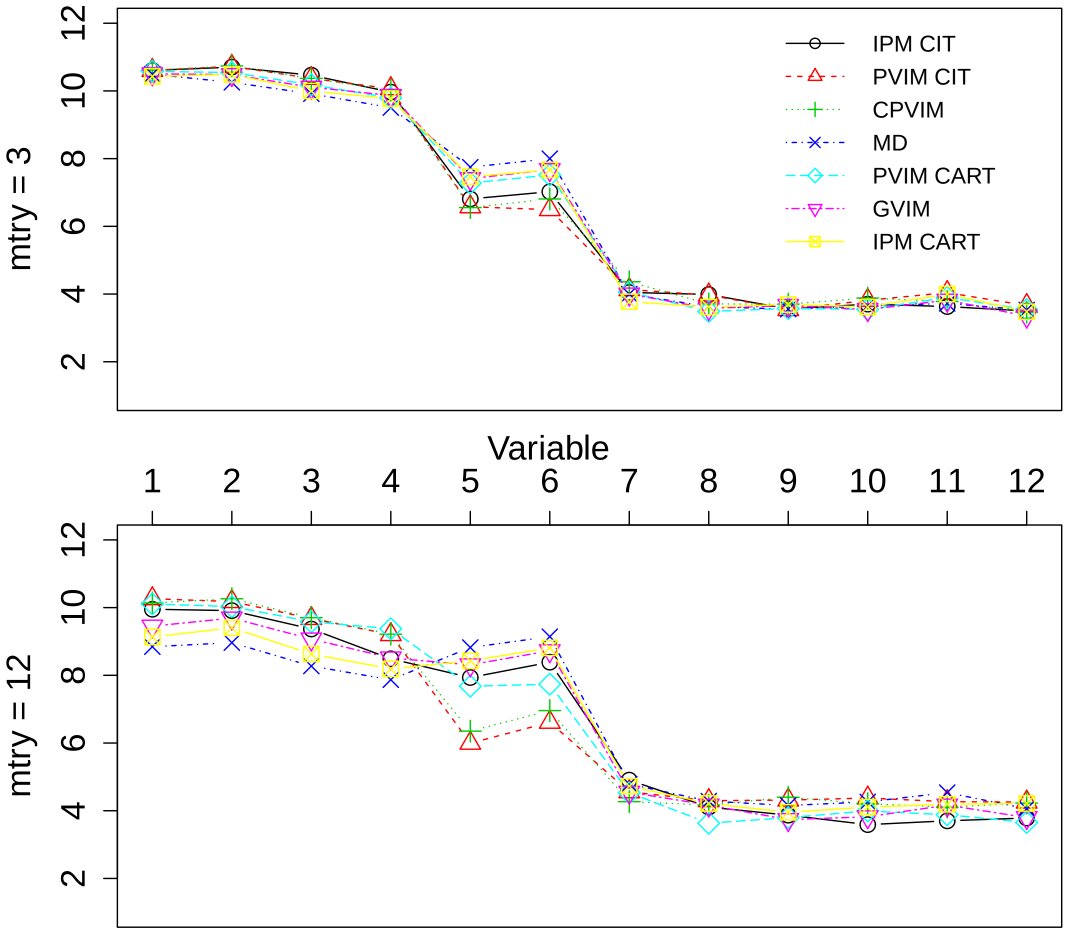

Figure S5: Average ranking for each VIM in Scenario 2, for $mtry$ =3 and $mtry$ =12, with n = 50. The code of each VIM appears in the figure legend.

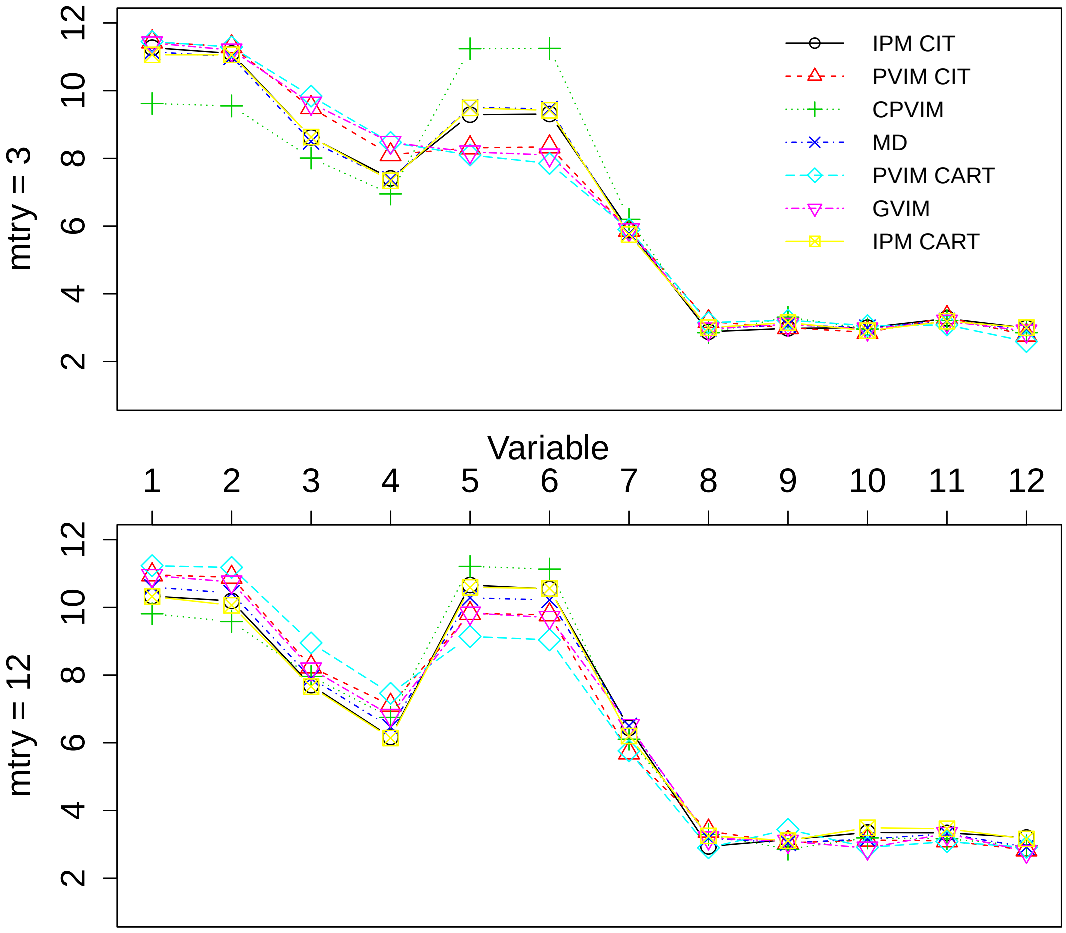

Figure S6: Average ranking for each VIM in Scenario 2, for $mtry$ =3 and $mtry$ =12, with $n = 500$. The code of each VIM appears in the figure legend.
